# Short-term creep approach to redefining the role of 17-4PH stainless steel for high-temperature applications

**DOI:** 10.1038/s41598-024-58273-7

**Published:** 2024-04-09

**Authors:** S. Spigarelli, M. Cabibbo, A. Santoni, E. Santecchia

**Affiliations:** DIISM-Università Politecnica delle Marche, Via Brecce Bianche 12, 60131 Ancona, Italy

**Keywords:** Short-term creep, 17-4PH, Constitutive equations, Microstructure, Particle-strengthening, Mechanical engineering, Metals and alloys

## Abstract

The creep response of the 17-4PH martensitic age-hardening steel in H1150 state was investigated at 427 and 482 °C. Hardness measurements of the heads of the creep samples demonstrated that the material underwent additional age hardening during the high-temperature exposure. Microstructural investigations confirmed that the additional precipitation of carbides and the G-phase occurred at the lowest temperature. A set of constitutive equations previously developed to describe the creep response of particle-strengthened alloys was successfully used to obtain a comprehensive description of the experimental data. The value of the particle strengthening term was obtained from the hardness measurements and corresponded to the Orowan stress. The model accurately described the observed minimum creep rate dependence on the applied stress and explained the occurrence of lower values of the minimum strain rate observed during variable-load experiments.

## Introduction

17-4PH (AISI 630) martensitic stainless steel is an age-hardening material with extensive industrial applications owing to its excellent mechanical properties and high corrosion resistance. Because this steel is hardened by the precipitation of secondary phases, it is hardly surprising that most of the available literature deals with its aging kinetics. In recent years, experimental studies have dealt with the properties of 17-4PH steel produced by additive manufacturing; however, these investigations will not be considered here because of the significant differences in microstructure with wrought material. However, a large database of experimental information on the aging response of this material, produced using traditional technologies, can still be obtained. Steel is usually austenitized/solutioned above 1000 °C and aged between 482 (H900 state) and 621 °C (H1150 state) (some authors have also investigated the effect of long permanence at lower temperatures)^1,2^. After solubilisation, within the typical temperature range employed for the hardening (482–621 °C), precipitation of copper particles occurs during the aging process. Conversely, tempering of martensite yields only minor effects on hardness^3^. Hsiao et al. found that peak-aging at 480 °C occurs just after 1 h. In this condition, the lath martensite matrix contains a very high density of dislocations and copper precipitates are still coherent. Overaging at 620 °C for 4 h caused the formation of reversed austenite after extensive precipitation of copper-rich particles. Thus, the formation of reversed austenite is suggested to be related to the preexisting copper particles^3^. Yeli et al.^4^, on the other hand, provided a more articulate picture of the precipitation kinetics in this steel. At 480 °C, according to these authors, the precipitation sequence is divided in the following steps: i. segregation of CrN/NbN on dislocations and other matrix defects; ii. Cu- and Nb-rich precipitate formation, and iii. Cr-rich particle precipitation; and iv. Formation of (Mn, Ni, Si)-rich phase. After ageing at 590 °C, no Cr- or (Mn, Ni, Si)-rich precipitates were observed, in agreement with the prediction from the phase diagram. Overageing occurs at both temperatures for sufficiently long time of exposure. Although, at 480 °C, the hardening contribution from Cr-rich precipitates compensates for the softening due to Cu-rich particles coarsening. In an interesting effort to provide a self-consistent description of the effects of these aging phenomena, Mirzadeh and Najafizadeh^5^ modelled the hardening behaviour of 17-4PH by means of artificial neural networks.

Prolonged (14–19 years) holding at 300 °C after ageing at 610 °C for 4 h was found to cause an increase in the number and size of Cu-rich precipitates and Cr-rich precipitates^6^. According to Bai et al., this phenomenon results in an increase in hardness, which is a strengthening effect more related to Cr-rich precipitates than to Cu-rich particles. A much shorter (up to 32 h) holding time at temperatures between 200 and 700 °C causes significant softening of the material in the H900 state only when the temperature exceeds 500 °C^7^. The overaged microstructure of the material in H1150 state, on the other hand, exhibits a moderate softening only after exposure at 700 °C.

Another important issue investigated in detail is the strengthening of precipitates^8,9^. According to Sun et al.^8^, dislocation can either shear or by-pass (Orowan mechanism) the fine Cu precipitates produced by ageing at 480 °C. Ageing at 620 °C, on the other hand, results in coarser precipitates that can only be by-passed by the Orowan mechanism.

**Figure 1:**
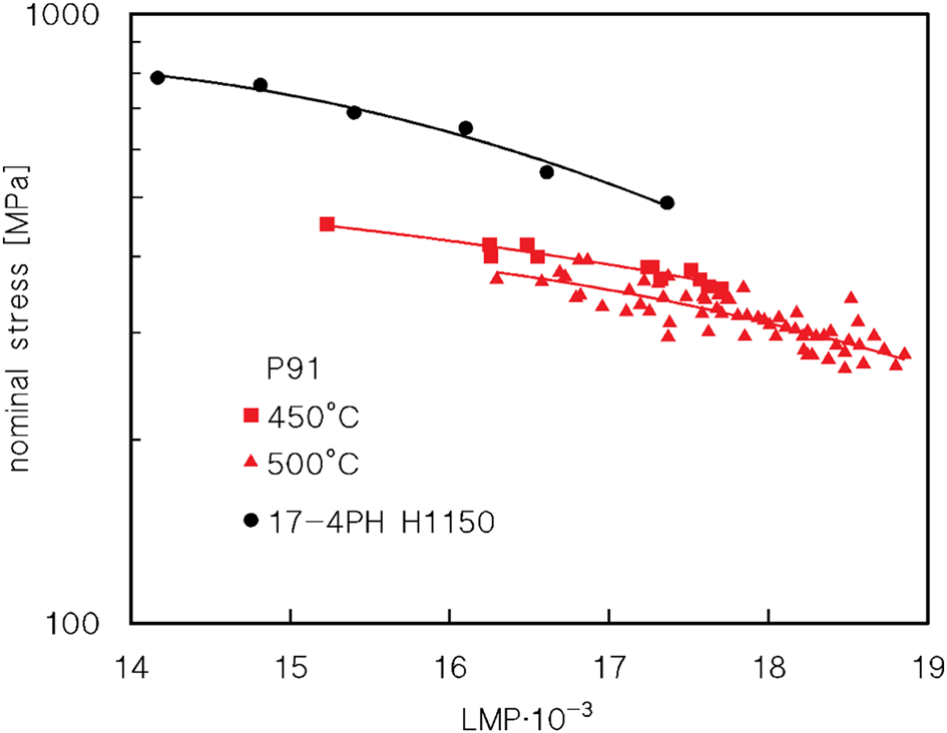
Comparison of P91 and 17-4PH H1150. Comparison between the alloy under investigation and the Larson-Miller parameter (LMP) vs. stress plot for P91 (data from Ref.^12^) and 17-4PH H1150^10^.

The few studies mentioned above are just a representative selection among the very large number of studies dealing with the relationship between the microstructure and room-temperature mechanical properties of 17-4PH. In this context, the complete lack of studies on the creep response of this steel is surprising. The only available dataset on the creep response of 17-4PH steel consists of a series of stresses for 100 and 1000 h of rupture provided by the material producer (ARMCO). These data are invariably reported in either books^10^ or material datasheets (see, for example^11^). In principle, one can argue that this lack of interest in the creep response is due to the intrinsic microstructural instability of age-hardening steel, which severely limits its potential applications at high temperatures. This reasoning leads to a simplistic view of the problem. Figure 1 plots the time to rupture (*t*_*R*_) vs stress (*σ*) values obtained at 450 and 500 °C of P91 steel (data from Ref.^12^), in form of Larson-Miller parameter (LMP), expressed as

1 $$LMP=T\left[20+{\text{log}}\left({t}_{R}\right)\right],$$

where *T* denotes absolute temperature. As the P91 steel is recognised worldwide as one of the standard materials for high-temperature (550–600 °C) applications^13^, this material can be considered a touchstone for determining if the 17-4PH is worthy of consideration for its creep response.

Figure 1 shows the few data reported in the abovementioned datasheet^10^ for the H1150 state (for temperatures between 370 and 482 °C). The stresses for a given LMP value were invariably much higher for 17-4PH than for P91 tested in a similar temperature range. Evidently, it is quite possible that longer exposure times lead to a progressive loss in creep strength (the two datasets seem to converge toward high LMP values). However, the evidence presented in Fig. 1 unambiguously demonstrates that the creep response of 17-4PH steel is worth studying in detail. The aim of this study was indeed to make up for this deficiency in material knowledge, by investigating the short-term creep response at 427 and 482 °C (800 and 1150 °F), providing also a simplified, easy-to-use, and reliable constitutive model for the minimum creep rate dependence on applied stress.

## Methods

### Material and samples

The 17-4PH steel (15 wt% Cr, 4 wt% Ni, 3 wt% Cu, 0.5 wt% Mn, Fe balance) investigated in this study was provided in the form of a bar of 17-4PH steel in a solutioned state. Dog-bone creep samples with 3 mm × 3 mm square sections and 25 mm gauge lengths were machined from the bar. After machining, the samples were heat-treated to the H1150 state (620 °C for 4 h). Heat treatment conditions were selected to obtain an over-aged and stable microstructure that was less susceptible to softening during creep at lower temperatures.

### Creep tests

The creep tests were performed using constant-load machines. Two types of experiments were carried out: (i) Constant-load experiments (CLEs), during which the initial load was maintained until rupture or interruption of the test. Variable load creep experiments (VLEs), in which the load was maintained at the initial value until the creep curve approached a constant creep rate, were then increased to obtain a second and higher value of the minimum creep rate at this higher stress. Table 1 summarises the results of creep experiments. The testing temperatures were 427 and 482 °C; these temperatures were chosen to permit a direct comparison with the information contained in Ref.^10^.

**Table 1:** Summary of experimental test matrix. The time to rupture does not include the soaking time (0.5 h).

Sample	T [°C]	Nominal stress [MPa]	Minimum strain rate [s^−1^]	Time to rupture [h]
VLE1	482	455	1.0 × 10^–9^	n.a.
		545	9.5 × 10^–9^	n.a.
VLE2		554	7.0 × 10^–8^	n.a.
		600	3.0 × 10^–7^	n.a.
VLE3		540	2.0 × 10^–9^	n.a.
		660	4.5 × 10^–7^	n.a.
CLE1		548	1.6 × 10^–8^	447
CLE2		550	8.1 × 10^–8^	161
CLE3		568	3.0 × 10^–7^	45
CLE4		600	5.6 × 10^–7^	21
CLE5		660	9.0 × 10^–6^	0.7
CLE6		700	3.6 × 10^–5^	0.2
VLE4	427	671	1.6 × 10^–9^	n.a.
VLE4		756	7.0 × 10^–8^	n.a.
CLE7		675	6.8 × 10–^9^	interr. 600
CLE8		678	2.6 × 10^–7^	49
CLE9		700	5.3 × 10^–7^	24
CLE10		710	3.9 × 10^–6^	3.8
CLE11		725	6.0 × 10^–6^	2.6

### HRC and aging response

The aging response of the alloy was investigated using Rockwell hardness (HRC) measurements of the sample heads. In this case, the exposure time included the soaking time before loading and the overall test duration, which included any permanence at the testing temperature after rupture.

The HRC measurements were converted into UTS values using a polynomial equation interpolating the data in the well-known ASTM A370/ASME SA-37. Only data in the range of 30–40 HRC (typical hardness values for the steel investigated in this study) were considered. The resulting equation is as follows:

$$UTS=0.07576{\left(HRC\right)}^{3}-7.15{\left(HRC\right)}^{2}+250.68\left(HRC\right)-2180.6 \left[{\text{MPa}}\right].$$

### TEM samples preparation

Thin foils for transmission electron microscopy (TEM) metallography were extracted from the crept sample heads. The samples were prepared by mechanical grinding and polishing to a thickness of ~ 110 µm, followed by dual-surface dimpling to a thickness of ~ 30 µm and finally ion-milled to electron transparency using a Gatan^©^ (Gatan Inc., Pleasanton, CA, USA) PIPS working at 8 keV (incident beam angle progressively reducing to 8°, 6°, and 4°). TEM analyses were performed using a Philips™ (Philips It, Milan, Italy) CM-200 microscope working at 200 kV and equipped with a double-tilt LN-cooled specimen holder. Selected area diffraction patterns (SAEDP) were recorded using a converged beam. Converged electron beam diffraction (CBED) was used to measure the thin foil thickness, *t*_*TEM*_, in the surrounding areas of detected phase particles. This was performed by analysing the corresponding diffracted beam intensity variation under dual beam conditions. Thence, linear interpolation of data points in a *S*^2^/*n*_*fringes*_^2^ vs. *n*_*fringes*_^−2^, where *S* is the fringes spacing, and *n*_*fringes*_ the number of counted fringes, was used to determine *t*_*TEM*_.

## Results

### Creep results

**Figure 2:**
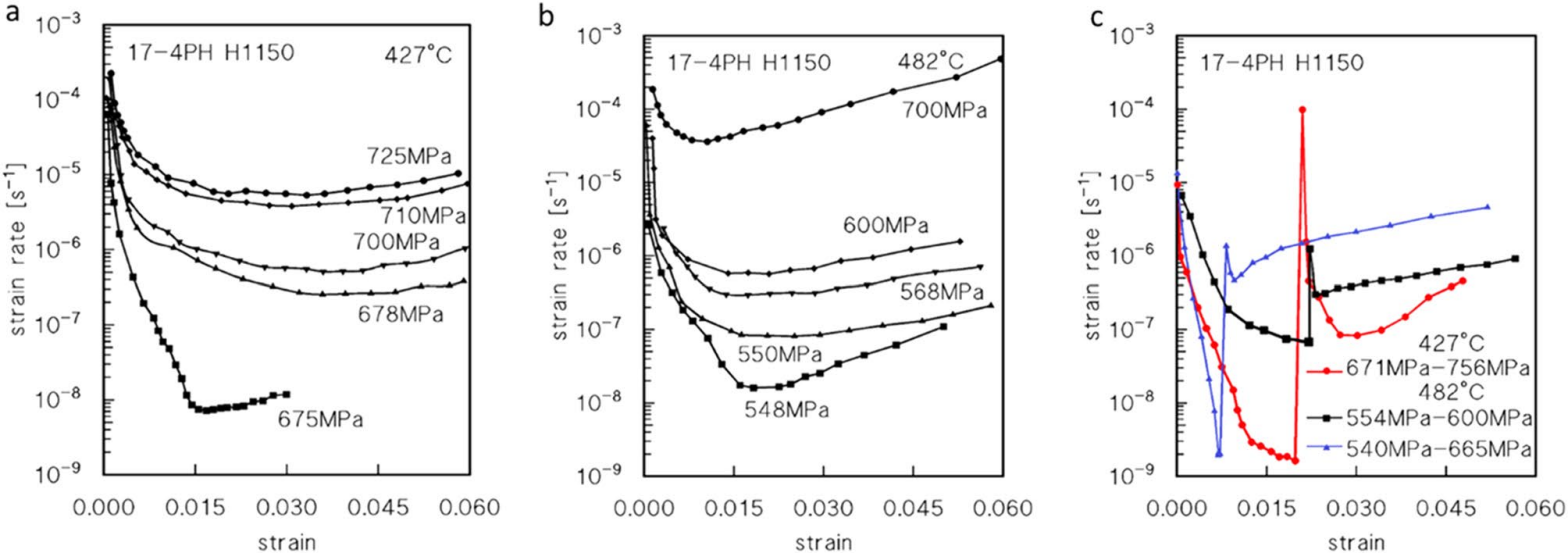
Strain rate vs. strain curves. Representative strain rate vs. strain curves for CLEs (**a,b**) and VLEs (**c**).

Figure 2 show the typical shapes of the creep rate versus strain creep curves. In general, the shape of the curves is conventional, with a short primary region, minimum creep rate range, and extended tertiary stage. Figure 2c, in particular, shows that at least the value of the minimum creep rate obtained in the VLE under 675 MPa at 427 °C, and 540 °C at 482 °C, could be somewhat overestimated, since the load was possibly increased before the true minimum creep rate range was attained.

**Figure 3:**
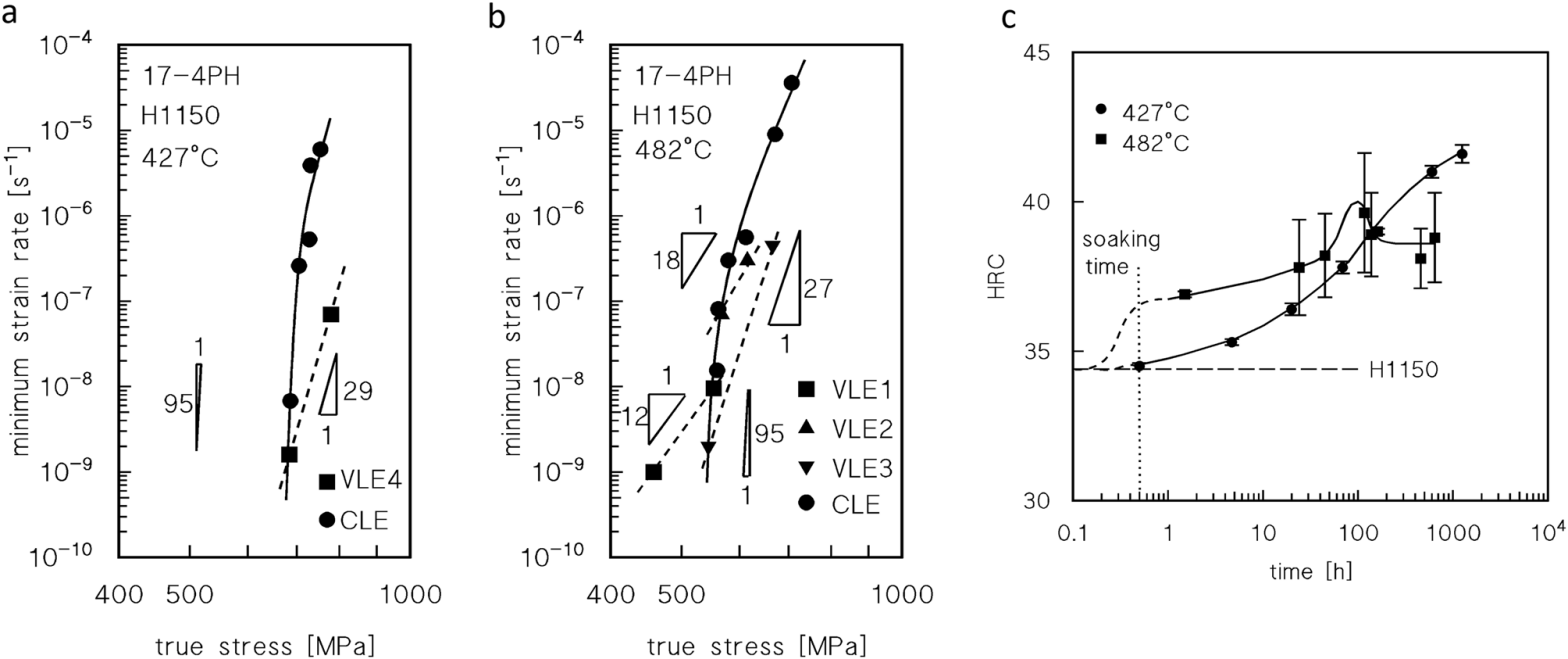
Minimum creep rate and hardness. Dependence on applied stress (true stress in correspondence of the minimum creep rate) (**a,b**), and HRC (samples head) as a function of total exposure at 427 and 482 °C (**c**). Broken lines in (**a,b**) connect the data obtained in the same VLE.

Figure 3a,b show the dependence of the minimum creep rate on true stress. The creep data roughly align the curves, whose slopes strongly increase with decreasing stress.

This behaviour is typical of particle-strengthened materials^14,15^. The datum obtained under the lowest stress at 482 °C substantially deviates from the curve, which suggests either that the minimum creep rate was overestimated by the VLE, or that the material underwent substantial softening due to long high-temperature exposure.

Interestingly, the data for the second loading in the VLEs deviate considerably from the curves obtained for the CLEs. In particular, the minimum creep rate observed after a load change is invariably much lower than the value obtained under the same load in the CLE. As a result, the slope of the straight line connecting the two minimum creep rate values obtained in each VLE, which ranges between 12 and 29, is substantially lower than the that observed in case of the whole curve in the same interval of strain rates. This evidence demonstrates that high-temperature exposure results in a substantial increase in the creep strength in most cases.

### Age hardening

Figure 3c shows the HRC values measured on the heads of the samples after the creep experiments as a function of the total exposure duration, which includes the soaking time at the testing temperature, the overall duration of the test, and the permanence at high temperatures after sample fracture. This figure is clearly representative of age-hardening behaviour. At the lowest testing temperature in the investigated range of experimental conditions, the HRC monotonically increased with time. At the higher testing temperature, a brief duration of 0.5 h is adequate to increase the material hardness by approximately 2 HRC. Subsequently, the HRC rapidly increases to a maximum and falls equally rapidly to an almost constant value.

The age-hardening response observed in the sample heads, as illustrated in Fig. 3, was not affected by the dislocation activity due to creep loading.

### Microstructural observations

**Figure 4:**
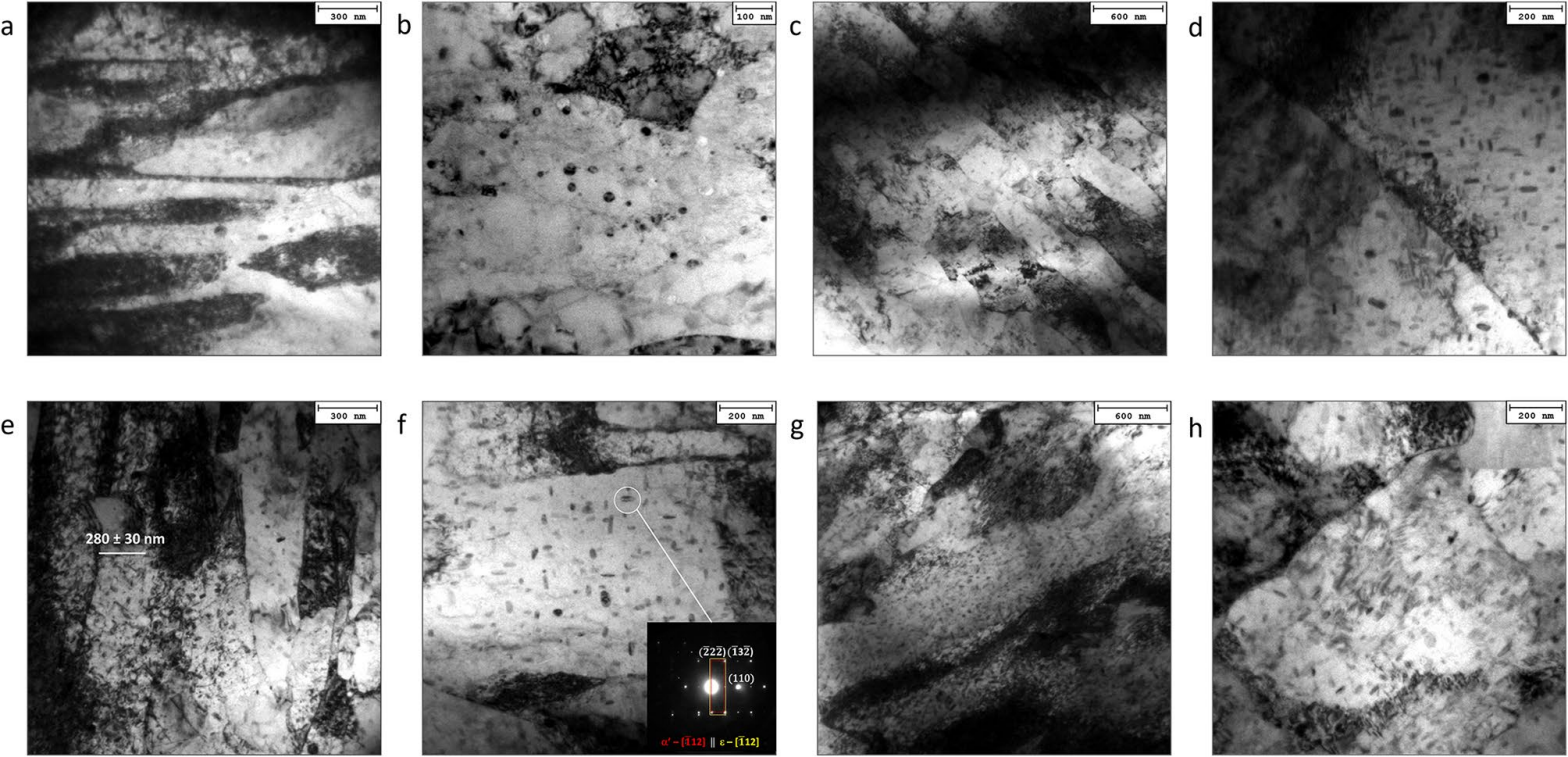
TEM investigation of the VLE1, VLE4 and CLE2 samples. Micrographs showing the typical martensite lath spacings and general view of the existing strengthening particles detected at the VLE4 sample heads in the H1150 bar (**a,b**); in the sample VLE4 tested at 427 °C (**c,d**). A significant number volume of rounded shaped oxides is shown in (**b**). Microstructure of the head of samples tested at 482 °C: VLE1, (**e,f**); CLE2, (**g,h**). Mean lath spacing is showed in (**e**); semi-coherency of ε-phase with αʹ matrix is documented by indexed SAEDP added to (**f**).

Figure 4 show the typical martensitic microstructure of steel after high-temperature exposure (sample heads).

The martensite phase consisted of a lath structure with a very high dislocation density. The presence of dislocation tangles within the martensite lath indicates a high stored energy. Mean lath spacing was measured as to be 0.8, 0.9, 0.9, and 1.1 µm in the as received material, in CLE2, VLE1, and VLE4 samples, respectively. The long temperature exposure did not significantly affect the martensite lath spacing.

By contrast, the as-received bar showed a significant number of dark round particles surrounded by dislocation tangles in some cases. These particles are identified as oxides. These oxide particles are seldom observed in the heat-treated samples.

The predominant phase formed at high temperatures was the ε-Cu-rich phase. According to the representative TEM micrographs in Fig. 4e, the ε particles appear to be mostly semi-coherent with the matrix. The interaction between this phase and the dislocations was found to be prevalent via a bypass-strengthening mechanism. The ε-Cu-rich particles represent the most effective nanometric strengthening phase under all the experimental conditions. These particles are typically elliptical-to-bar-shaped, with a mean equivalent diameter of a few nanometres to 30 nm. Murayama et al.^1^ reported the chemical composition of the ε-Cu-rich phase to be 55 at. pct Cu, 30 at. pct Fe, 10 at. pct Cr, and 5 at. pct Ni. The copper concentration in the fine ε Cu-rich particles that precipitated during tempering is in good agreement with an earlier study by Goodman et al.^16^.

**Figure 5:**
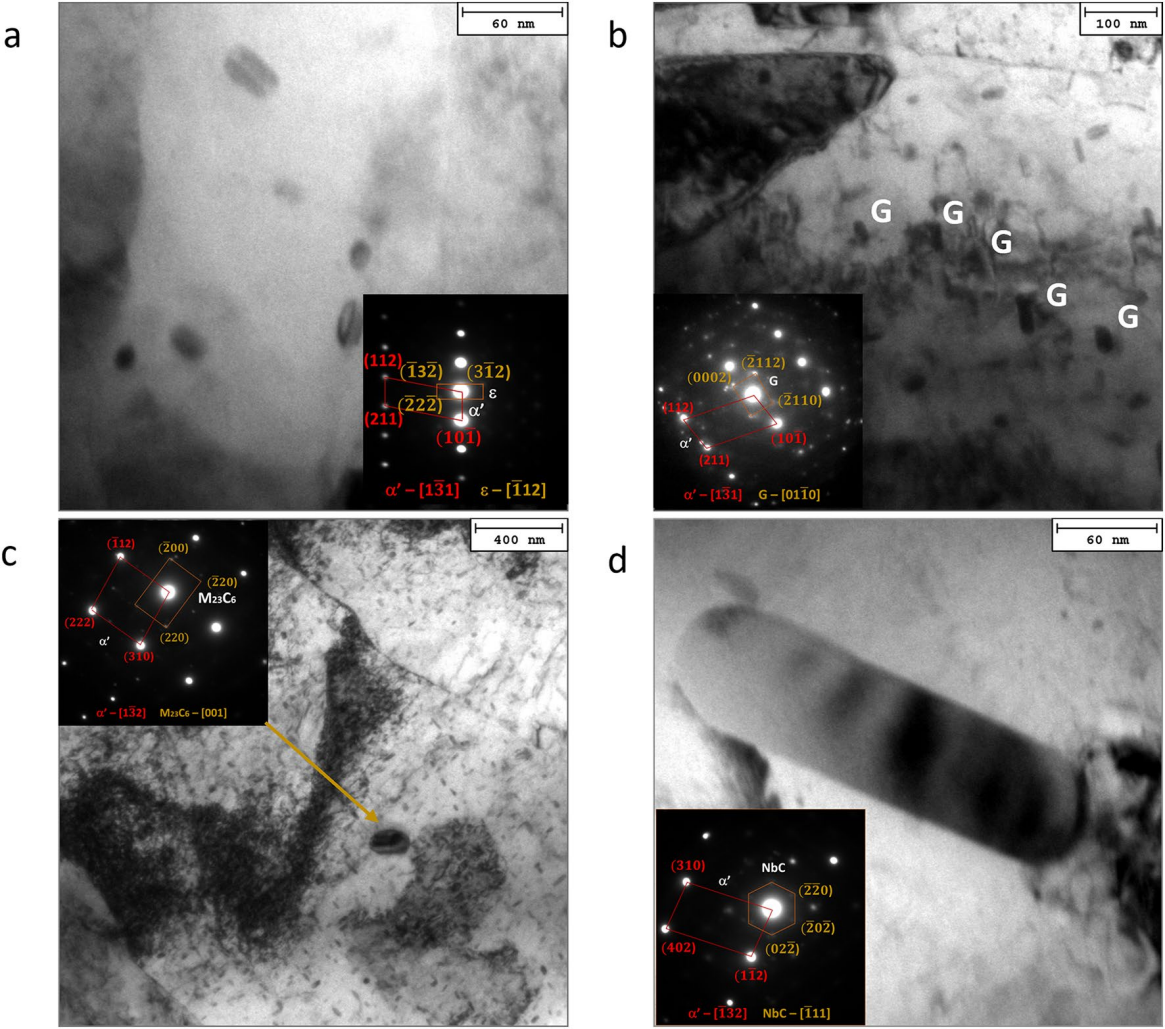
TEM results of the detected phases. Micrographs showing the detected phases with related indexed SAEDPs (VLE4); ε-Cu rich particles (**a**); G-phase (**b**); M_23_C_6_ carbides (**c**); NbC carbides (**d**).

With increasing aging time and temperature, fine spindle-shaped precipitates were observed and identified as the G-phase with an FCC crystal structure alongside the Cr-rich particles (Fig. 5).

The G phase is a Ni- and Si-rich intermetallic compound with a stoichiometric composition of Ni_16_Si_7_M_6_, where M represents Fe, Mn, V, and Nb^17^. Murayama et al. observed that small ε Cu-rich particles were always adjacent to the G-phase, suggesting that the ε phase was able to provide heterogeneous nucleation sites for G-phase precipitation^1,17^. This was confirmed by microstructural inspection. The G phase has a crystallographic structure of type D8a and space group Fm3m, which is isotypic with Th_6_Mn_23_, as reported in Refs.^2,18^.

**Figure 6:**
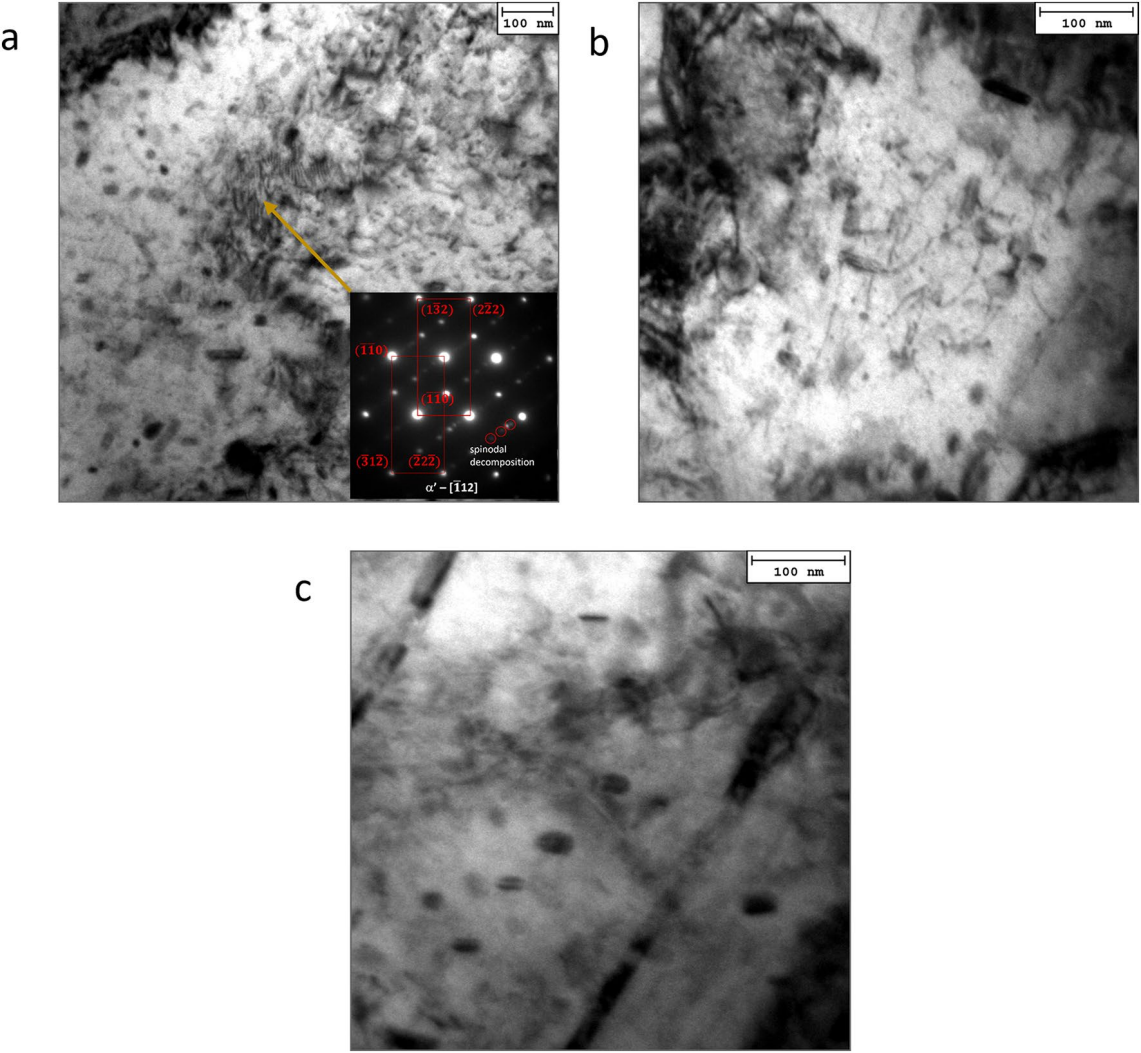
αʹ spinodal decomposition. Representative TEM micrograph αʹ spinodal decomposition occurring for long high-temperature exposure duration in VLE4 sample head: (**a**), with indexed SAEDP (inset); a detail of dislocation strengthening by ε-Cu rich nanoparticles is shown in (**b**), the semi-coherency of a large fraction of such ε particles is reported in (**c**).

A further feature induced by aging at the creep-testing temperature is the detected spinodal decomposition of the martensite phase, which can be recognised as the formation of fine-scale bright-dark fringes by which α-ferrite decomposes to form a Cr-rich αʹ-phase (bright fringes) alternated by the Fe-rich αphase (dark fringes) (Fig. 6).

These were generally observed at and along the grain boundaries, and the α and αʹ phases exhibited the same crystal structure. Ramanarayan et al.^18^ proposed that spinodal decomposition occurs at the grain boundaries because these regions of the steel matrix have higher free energy. Thus, these regions have higher atomic mobility. This implies that phase decomposition is induced in the martensite phase by long permanence at high temperatures, and the martensite decomposes into Fe-rich and Cr-enriched α phases.

As the high-temperature aging time increased, the ε-Cu-rich particles coarsened by a typical Ostwald ripening mechanism, leading to softening (see the HRC variations at 482 °C). In the case of the creep head sample maintained at 427 °C for the longest duration (VLE4, Fig. 6), the expected softening due to ε particle ripening was more than compensated for by the further induced precipitation of different phases, such as the G-phase and NbC carbides. Both phases consist of nanometric particles which act as effective barriers to dislocation mobility. Both spinodal decomposition and the formation of fine Cr-rich secondary carbides (M_23_C_6_) were important strengthening contribution features that were activated after the longest high-temperature exposure (Fig. 5). This phenomenon can be explained by the redistribution of atoms, mainly Cu and Cr, followed by the progressive formation of the G phase, M_23_C_6_, and α spinodal decomposition^19^.

In recent years, it has been shown that the *G*-phase exists in various Fe–Cr–Ni alloys as a dispersed phase in the interior of grains^20–22^. Auger et al.^22^ reported phase separation and precipitation of the *G*-phase in the ferrite phase of duplex stainless steel. They concluded that the nucleation of the *G*-phase takes place at the ‘‘interface’’ between Cr-rich aʹ and Fe-rich a. For 17-4PH stainless steel, Wang et al.^23^ found that *G*-phase precipitation occurs in intimate contact with ε-Cu precipitates following spinodal decomposition. This result confirmed that the Cu precipitates provided heterogeneous nucleation sites for *G*-phase formation. One reason for this is that the Cu/interface is a more suitable site for the nucleation of the *G*-phase than the α/αʹ interface.

Table 2 reports the statistical data of all the detected particles under the four different experimental conditions inspected by TEM, that is, particle volume fraction, *f*_*V*_, spacing, *λ*, mean equivalent diameter, *d*_*eq*_, and the occurrence of their formation induced by the high-temperature baking temperature and duration.

**Table 2:** Statistic evaluation of secondary phase particles and carbides formed during high temperature and time exposition of the creep-sample heads.

	f_V_, %	λ, nm	d, nm	ε-Cu rich	G-phase	M_23_C_6_	NbC
As received	5 ± 1	400 ± 10	31 ± 3	x	–	–	–
CLE2	10 ± 2	138 ± 6	23 ± 2	x	–	x	–
VLE1	11 ± 2	147 ± 6	27 ± 2	x	–	x	x
VLE4	9 ± 1	190 ± 8	29 ± 2	x	x	x	x

No distinction was made between the different families of particles in calculating the total volume fraction, the spacing and the average size.

## Discussion

### Orowan strengthening in overaged steel at room temperature

The hardness variations with time and temperature can be described as the net result of strengthening by α spinodal decomposition, precipitation of the G-phase and M_23_C_6_ carbides, and softening due to Ostwald ripening of the ε-Cu-rich precipitates. Effect of microstructural evolution on the hardening behaviour of 17-4PH over the long term 427 to 482 °C ageing can be thus summarized as follows:

i. The initial microstructure after solution treatment and tempering (aging) consisted of lath martensite and nanosized ε Cu-rich particles. As the high-temperature exposure of the steel is prolonged or the temperature increases, α–αʹ forms via spinodal decomposition along the grain boundaries.
ii. With further high-temperature exposure, the precipitation of G-phase particles and carbides, such as M_23_C_6_ and NbC, occurred, providing additional strengthening. This leads to rapid hardening despite the expected softening owing to concurrent ε particle ripening.

Dislocations can negotiate with coherent particles by shearing or bowing. Small coherent particles can be cut more easily, whereas larger particles must be bypassed using the Orowan mechanism^6,8,9^. Evidence of the presence of dislocation loops around ε-copper particles led Sun and co-workers to conclude that after ageing at 620 °C (H1150 state), the precipitates could be more easily bypassed by the Orowan mechanism. Thus, the particle-strengthening term *σ*_*p*_ can be assimilated to the Orowan stress, which, according to its simplest formulation, can be expressed as^24,25^

2 $${{\sigma }_{p}=\sigma }_{Or}=0.84\frac{MGb}{{\uplambda }_{s}}.$$

*G* is the shear modulus, *b* is the length of the Burgers vector, *M* is the Taylor factor (here assumed = 3), and *λ*_*s*_ is the surface-surface distance between particles, expressed as *λ-d*. Assuming the vast majority of the strengthening particles to be Cu-rich precipitates, and putting *d* = 23 and 27 nm, λ = 138 and 147 nm for the samples tested under 550 MPa (CLE2, total time of exposure 161 h, HRC = 39) and 455/545 MPa (VLE1, total time of exposure 642 h, HRC = 38.8) at 482 °C, respectively, Eq. (1) yields *σ*_*Or*_ ≈ 450 and 445 MPa. The tensile strengths of these samples, as obtained from HRC measurements, were ≈ 1220 and 1210 MPa, respectively. Subsequently, we assume that the *UTS* of the material can be calculated by an equation in the form of (3):

3 $$UTS={UTS}_{m}{+ \sigma }_{p}={UTS}_{m}{+ \sigma }_{Or},$$

where *UTS*_*m*_ is the tensile strength of the annealed matrix. An approximate calculation suggests that *UTS*_*m*_ should be in the range of 765–770 MPa, which is consistent with the typical UTS values of annealed high-Cr martensitic steels^26^. This value includes all typical strengthening mechanisms operating at room temperature in tempered martensite (dislocation hardening, solid solution strengthening, etc.). The *UTS* of annealed high-Cr steels is reasonably close to the *UTS*_*m*_ value estimated by Eq. (3), suggesting that in these samples Orowan by-pass is likely to play the role of main particle-strengthening mechanism, at least for the H1150 state, which represents the steel “overaged condition”.

### Quantification of the particle strengthening term at room temperature for various time of exposures at 427 and 482 °C

**Figure 7:**
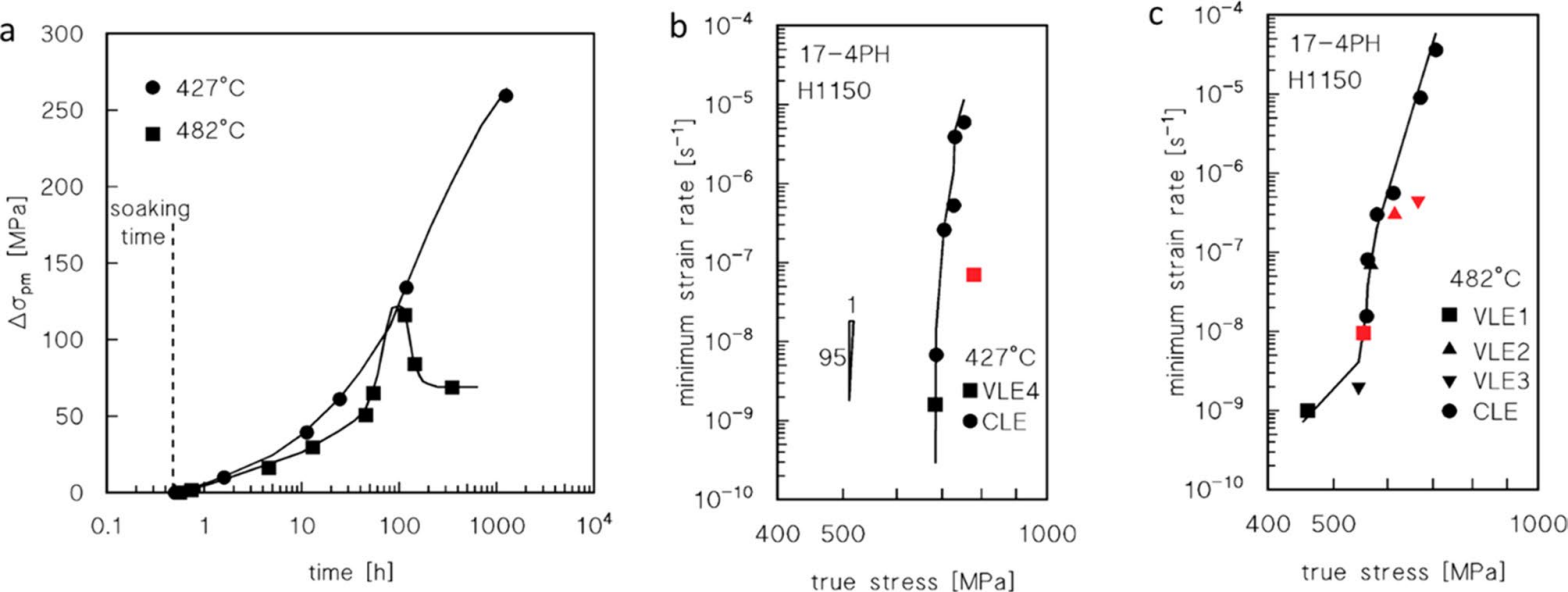
Strength dependence on minimum creep rate and model curves. Increase in strength in correspondence of the minimum creep rate, with respect to the HRC value at the test temperature just before loading (**a**). Model curves calculated for CLEs and data from first step of VLEs (**b,c**). Data-points in red represent the minimum creep rate measured during the second step of the VLEs.

The *HRC* vs. time plots presented in Fig. 3c can be used to estimate the strengthening role of precipitates after various exposure times. The aging curves in the figure, after 0.5 h of exposure (soaking time) at 427 and 482 °C, give HRC ≈ 34.6 and 36.6 respectively (UTS ≈ 1080 and 1125 MPa). At *UTS*_*m*_ = 765 MPa, the resulting particle-strengthening terms (Orowan stress) are *σ*_*Or*_ ≈ 340 and 320 MPa (*λ* ≈ 170 and 180 nm, respectively, and *d* = 20 nm). This condition represents the state of steel immediately prior to loading. The curves in Fig. 3 can be then used to roughly estimate the particle-strengthening term after different times of exposure at high-T, by assuming that the variation in hardness (and *UTS*) only depends on the variation of *σ*_*p*_, i.e., *UTS*_*m*_ at room temperature does not vary appreciably with time of exposure at 482 and 427 °C. This assumption makes sense because the ageing/tempering was carried out at much higher temperatures, and the recovery of the initial martensitic structure had already occurred during that treatment. Figure 7 plots the increase of the particle strengthening term at room temperature, obtained by HRC measurements, i.e., Eq. (4):

4 $$\Delta {\sigma }_{pm}={\sigma }_{pm}-{\sigma }_{p0},$$

where *σ*_*pm*_ and *σ*_*p0*_ are the *σ*_*p*_ values at time *t* = *t*_*m*_ and after soaking (0.5 h) at *T* before loading, respectively.

### Constitutive equations for particle-controlled creep

The model considered herein is a derivation of constitutive equations originally developed by Sandström^27–29^. This model is based on the well-known Taylor equation in the form of (5):

5 $$\sigma \approx {\sigma }_{p}+{\sigma }_{\rho }={\sigma }_{p}+ {\alpha }_{0}mGb\sqrt{\rho },$$

where *ρ* is the dislocation density, *α*_*0*_ is a constant and *σ*_*ρ*_ = α*mGbρ*^1/2^ is the dislocation hardening term. The strengthening effect due to interaction between particles and dislocations is expressed by the *σ*_*p*_ term. In this simplified form, the model did not consider solid-solution strengthening, nor any additional strengthening mechanisms, such as spinodal decomposition.

The evolution of dislocation density during straining can be expressed as Eq. (6)^27–29^:

6 $$\frac{d\rho }{d\varepsilon }=\frac{m}{bL}-\frac{2}{\dot{\varepsilon }}{M}_{cg}{\tau }_{l}{\rho }^{2},$$

where*τ*_*l*_ is the dislocation line tension (*τ*_l_ = 0.5*Gb*^2^), *M*_*cg*_ is the dislocation mobility, and *L* is the distance travelled by a dislocation before it undergoes a reaction (dislocation mean free path).

In materials with a distribution of densely spaced particles, the following relationship can hold (Eq. 7):

7 $$\frac{1}{{\lambda }_{s}}\approx \frac{1}{L}.$$

At the steady state or in the minimum creep rate range, a combination of Eqs. (5)–(7) gives Eq. (8):

8 $${\dot{\varepsilon }}_{m}=\frac{2{M}_{cg}{\tau }_{l}b{\lambda }_{s}}{m}{\left(\frac{{\sigma }_{\rho }}{{\alpha }_{0}mGb}\right)}^{4}.$$

Dislocation mobility can be written as Eq. (9)^30^:

9 $${M}_{cg}\cong \frac{{D}_{0L}b}{kT}{\text{exp}}\left(\frac{{\sigma }_{\rho }{b}^{3}}{kT}\right){\text{exp}}\left\{-\frac{{Q}_{L}}{RT}\left[1-{\left(\frac{{\sigma }_{\rho }}{{R}_{max}}\right)}^{2}\right]\right\},$$

where *R*_*max*_ represents the maximum alloy strength, which is roughly quantified as 1.5 times the *UTS* at the testing temperature, *R* is the gas constant, *k* is the Boltzmann constant, and Eq. (10)

10 $${D}_{L}={D}_{0L}{\text{exp}}\left(-\frac{{Q}_{L}}{RT}\right),$$

denotes the self-diffusion coefficient. For iron, *D*_*0L*_ = 3.71 × 10^–4^ m^2^ s^−1^, *Q*_*L*_ = 269 kJ mol^−1^^31^, *b* = 2.5 × 10^–10^ m^32^, while the shear modulus was obtained from Ref.^33^.

The constitutive equations introduced above require only the *λ*_*s*_ and *σ*_*pm*_ terms as microstructural input data to estimate the minimum creep rate at a given stress. The particle strengthening term at room temperature, the Orowan stress, is already available (Fig. 7a, Eq. 4); the relevant value at high temperature can be simply estimated as Eq. (11):

11 $${\sigma }_{pm}={{\sigma }_{pm}}^{RT}\frac{G}{{G}_{RT}}={\left({\sigma }_{p0}+\Delta {\sigma }_{pm}\right)}^{RT}\frac{G}{{G}_{RT}},$$

where the *RT* subscript/superscript indicates the relevant value at room temperature.

The interparticle distance in correspondence of the minimum creep rate *λ*_*sm*_ can be tentatively calculated as Eq. (12):

12 $${\lambda }_{sm}=0.84\frac{MGb}{{\sigma }_{pm}},$$

which is formally correct whenever the particle strengthening term is equivalent to the Orowan stress in Eq. (1). Taking *α*_0_ = 0.3, and *R*_*max*_ = 1128 and 1200 at 482 and 427 °C, corresponding to a *UTS* at temperatures of 750 and 800 MPa, respectively (17-4PH datasheet^10,11^), the model curves presented in Fig. 7b,c were obtained. The model accurately captured the typical curvature of strain rate versus stress plots. In addition, the reduction in slope observed in the low-stress region at 482 °C is correctly described by the model, which confirms that the experimental value of the minimum creep rate is substantially correct.

**Figure 8:**
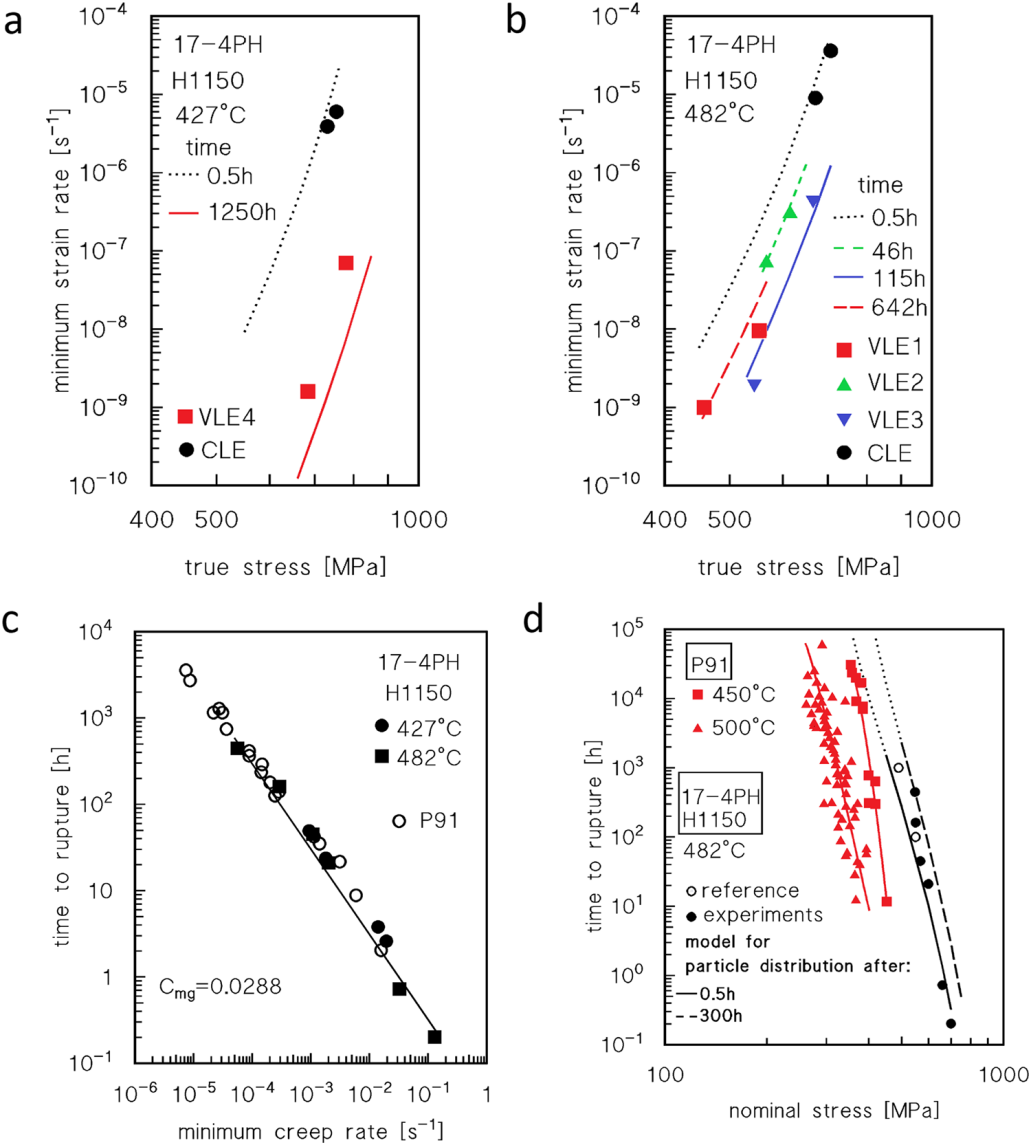
Model curves calculated for different times of exposure, Monkman–Grant and time to rupture vs. nominal stress. Model curves calculated for different times of exposure (**a,b**); for each curve, the strengthening effect of the particles was assumed to be equivalent to that calculated for the reported time of exposure. The VLE data roughly align on the curve corresponding to the relevant time of exposure, which is correct, because the two values of the minimum creep rate for any VLE were obtained for times of exposure that had a minimal difference. (**c**) Monkman–Grant plot of the time to rupture as a function of minimum strain rate. The straight line in the figure was purposedly selected to provide a conservative estimate of the time to rupture (experimental values are in some cases 1.5–2 times larger than that calculated from the curve). For comparison purposes, the data for P91 from Ref.^13^ are also reported; (**d**) plot of time to rupture (h) vs nominal applied stress for the steel investigated in the present study. For comparison purposes, the data for 17-4PH steel from Ref.^10^ and for P91 from Ref.^12^ are also reported. Dotted curves indicate extrapolations to be carefully considered.

Figure 8a,b shows the model curves obtained for different values of the time of exposure: in case of the VLEs, the time of exposure corresponds to the average of the two *t*_*m*_ values, which had minimal differences. In addition, the minimum creep rates for the shortest CLEs are included in the figure because the overall exposure time corresponding to the minimum creep rate did not differ appreciably from 0.5 h. In this case the model curves are almost straight lines, over which the experimental values of the minimum strain rates for VLEs at 482 °C approximately overlap.

This evidence demonstrates that the simplified model presented here can describe the peculiar material response with reasonable accuracy, thus demonstrating the key effects of hardening and softening phenomena during creep aging. At 427 °C, the description is somewhat less accurate. This fact seems to suggest that extrapolation of the particle hardening contribution at high temperatures, roughly quantified by hardness measurements at room *T*, overestimates the strengthening effect of the various families of precipitates, which include the G-phase. In fact, a *σ*_*p*_ value just 40 MPa lower (450 MPa instead of 490 MPa at 427 °C) provides an excellent description of the minimum creep rates measured in the VLE4 test. At room temperature, this value corresponds to a UTS value approximately equal to that of 1300 MPa, that is, a hardness of 41 HRC, which is well within the range of experimental values (40–43 HRC).

Conversely, Table 2 indicates that the interparticle spacing after long-times of exposure at 427 °C is larger than that predicted by the model through Eq. (12). This suggests that, at least at room temperature, the use of the average interparticle spacing in the Orowan equation causes an underestimation of the particle-strengthening contribution. Moreover, even the high dislocation density introduced by the high stresses could play a role in altering the precipitation and softening phenomena. Conclusively, the model discussed here catches the overall material response adequately, even though it provides an oversimplified description of the hardening phenomena at 427 °C.

### Can the 17-4PH steel be considered for medium-temperature creep applications?

The fact that 17-4PH steel could be a candidate for high temperature applications worthy of consideration, at least in the range between 400 and 500 °C, was apparent in Fig. 1. The previous sections demonstrated that the material response can be properly described by a model which primarily considers the strengthening effect of the precipitates if the case is properly tuned to consider the role of different families of particles at lower temperatures. Thus, one can legitimately ask whether the method can be used to predict the most important parameter in the design of high-temperature equipment, that is, the time to rupture (*t*_*r*_). Any extrapolation of the material response based on the short tests discussed herein must be considered carefully. Nevertheless, one can start the analysis from the Monkman–Grant equation, Eq. (13):

13 $${\dot{\varepsilon }}_{m}{t}_{r}={C}_{mg},$$

where *C*_*mg*_ is a constant (Fig. 8c). The straight line shown in the figure was selected to provide a conservative estimate of the time to rupture, thereby avoiding overly optimistic evaluations of the material response. In this range of experimental conditions, the short-term data for 17-4PH and P91 largely overlap.

The combination of Eqs. (12) and (13) yields the two curves shown in Fig. 8d. Both these curves were calculated for the same temperature, 482 °C, by supposing that the particle size and distribution did not change during the test. One curve was then calculated by assuming that the particle size and distribution were the same as those observed after a 0.5 h permanence at 482 °C (that is, the softer condition considered in Figs. 7, 8). The second curve was calculated by assuming that particle size and distribution were comparable with those observed after 300 h at 482 °C (overaged condition in Fig. 7). The dotted curves represent the extrapolation of the model curves for a long exposure time, which as obvious cannot, in itself, provide a reliable estimation of the mid-term response, due to the lack of data in this region. The analysis of the figure shows how the experimental values of the time to rupture overlap with the first curve for high stresses (low durations) but progressively move toward the second curve as the stress decreases. Interestingly, the “reference data”^10,11^ are intermediate between the two model curves. A possible explanation for this is that over-aging results in a further reduction in the strengthening effect of the particles as time increases. Thus, the material response gradually approaches the curve corresponding to the softer condition (solid line in Fig. 8d). Finally, the data for P91^12^ for durations of up to 1000 h were well below the model curve for the softer state of 17-4PH. Thus, we confirmed that 17-4PH deserves further investigation to properly assess its potential as a creep-resistant material, in particular for tests of duration well above 1000 h. Information in this regard, as mentioned in the Introduction, is not available, at least from openly available sources.

## Conclusions

The present study investigated the short-term creep response at 427 and 482 °C of the 17-4PH stainless steel. The experimental results demonstrated that the steel exhibited the typical behaviour of a particle-strengthened alloy. High-temperature exposure after the H1150 heat treatment resulted in additional precipitation and overaging at the highest temperature. Microstructural investigation revealed the presence of different families of precipitates, i.e., ε-Cu rich particles and carbides at 482 °C, and ε-Cu rich particles, carbides, and G-phase precipitates at 427 °C.

The dependence of the minimum creep rate on applied stress and temperature was described using a constitutive model recently developed for particle-strengthened alloys. The introduction of a particle strengthening term directly correlated with the hardness increment experienced during aging, resulting in an excellent description of the experimental data at the highest temperature. At 427 °C, the model overestimated the particle-strengthening effect. Nevertheless, the general trend of the minimum creep rate was well described. The analysis of the time to rupture dependence on applied stress revealed that the 17-4PH steel, in the investigated range of experimental conditions, is more performant that one of the most praised steels for high-temperature applications, the P91. Although microstructural instability, in particular overageing, can impair its long-term resistance to some extent, the available data confirm that 17-4PH steel is worthy of greater attention and renewed studies on its creep response.

## Data Availability

The data that support the findings of this study are available from the corresponding author upon reasonable request.
